# Participation in a trial in the emergency situation: a qualitative study of patient experience in the UK WOLLF trial

**DOI:** 10.1186/s13063-018-2722-4

**Published:** 2018-06-25

**Authors:** Elizabeth Tutton, Juul Achten, Sarah E. Lamb, Keith Willett, Matthew L. Costa, on behalf of the UK WOLLF Research Collaborators, Julie Bruce, Sonia Davis, Susie Hennings, Stavros Petrou, Steven Jeffery, Damian Griffin, Benjamin Parker, James Masters, Nick Parsons

**Affiliations:** 1 Warwick Research in Nursing, Warwick Medical School, University of Warwick, Gibbet Hill Campus, Coventry, CV4 7AL UK; 2 Kadoorie Centre, Oxford University Hospitals NHS Foundation Trust, John Radcliffe Hospital, Oxford, OX3 9DU UK; 3 Clinical Trials Unit, Warwick Medical School, University of Warwick, Gibbet Hill Campus, Coventry, CV4 7AL UK; 4 Nuffield Department of Orthopaedics, Rheumatology & Musculoskeletal Sciences, University of Oxford, Oxford, OX3 9DU UK; 5 University Hospitals Coventry and Warwickshire NHS Trust, Clifford Bridge Road, Coventry, CV2 2DX UK

**Keywords:** Qualitative, Interviews, Experience, Consent, Emergency, Trial

## Abstract

**Background:**

Patients can struggle to make sense of trials in emergency situations. This study examines patient experience of participating in the United Kingdom, Wound management of Open Lower Limb Fractures (UK WOLLF) study, a trial of standard wound management versus Negative Pressure Wound Therapy (NPWT).

**Methods:**

The aim of the study was to understand the patient’s lived experience of taking part in a trial of wound dressings. Interviews drawing on Phenomenology were undertaken with a purposive sample of 20 patients, on average 12 days into their hospital stay from July 2012–July 2013.

**Results:**

The participants were vulnerable due to the emotional and physical impact of injury. They expressed their trial experience through the theme of being compromised identified in categories of being dependent, being trusting, being grateful and being without experience. Participants felt dependent on and trusted the team to make the right decisions for them and not cause them harm. Their hopes for future recovery were also invested within the expertise of the team. Despite often not being well enough to consent to the study prior to surgery, they wished to be involved as much as possible. In agreeing to take part they expressed gratitude for their care, wanted to be helpful to others and considered the trial interventions to be a small component in relation to the enormity of their injury and broader treatment. In making sense of the trial they felt they could not understand the interventions without experience of them but if they received NPWT they developed a strong technological preference for this intervention.

**Conclusions:**

Patients prefer to be involved in studies within the limits of their capacity, despite not being able to provide informed consent. A variety of sources of knowledge may enable participants to feel that they have a better understanding of the interventions. Professional staff need to be aware of the situated nature of decision making where participants invest their hopes for recovery in the team.

**Trial registration:**

Current Controlled Trials, ID: ISRCTN33756652. Registered on 24 February 2012.

## Background

The decision to take part in clinical trials in emergency circumstances can be problematic, patients are often in psychological shock, have pain and are receiving medication that affects their ability to think clearly; for example, opiate painkillers [1]. For this reason trials in emergency settings often use consent waivers where individual consent is not required, or deferred consent inviting participants to consent to continue in the study after randomisation. Deferred consent using verbal consent prior to randomisation with written consent post randomisation may be used but staff can find undertaking randomisation without a signed consent form difficult [2]. In the UK WOLLF trial if a patient lacked capacity a personal or nominated consultee was asked to advise the team regarding the patient’s participation in the trial, and patient consent was gained for continued enrolment in the study when they were well enough [1].

Understanding patient experience is an important part of patient-based evidence [3] and can be used as a basis for shared decision making between professionals and patients [4]. Current evidence provides insights into why patients chose to take part in trials and what the benefits and challenges might be for them. However, there is a gap in knowledge about how trials impact on patients who have experienced traumatic musculoskeletal injury. To explore this further, two linked studies were undertaken. The first was to examine patient experience of traumatic injury, in this case open-fracture of the lower limb. This experience is reported separately [5, 6]. The second is reported here and describes the patient experience of being part of a trial in the context of emergency interventions. The vehicle for this was the United Kingdom, Wound management of Open Lower Limb Fractures (UK WOLLF) a trial of standard wound management versus Negative Pressure Wound Therapy (NPWT) [5, 7]. In order to understand trial participation within their overall experience, a methodological approach was used that enabled patients to present their experience of what it was like being in a wound-dressing trial in emergency circumstances. How they made sense of the trial was situated in relation to other aspects of their experience. This patient-informed evidence could be used in the education of future practitioners involved in consenting patients to emergency trials in musculoskeletal injury.

Previous studies demonstrate a range of reasons for taking part in trials, benefits and challenges. Altruism is often identified by patients as important [8–10] alongside other considerations such as: avoiding surgery and the risk of infection [11], increased surveillance and continuity of care or the opportunity to have a new treatment [12], their health and personal implications of taking part [9]. Understanding trial design can be challenging and therapeutic misconception can occur where patients, despite being in a trial, feel their treatment was tailored to their specific needs [8, 11–13]. ‘Subjects manifest a therapeutic misconception when they fail to appreciate the risks and disadvantages of participating that are inherent in the research design’ and which may limit their care [14]. In a trial focussed on ankle injury participants felt a preference for one of the treatments when consenting but later felt that they had the best treatment for them. The treatment had become a normal part of everyday life, despite the participants knowing it was randomly allocated. In addition they indicated that in making a decision to take part they could not ‘know’ the treatments as they had not experienced them. This suggests that experiential knowledge is important to how participants make sense of studies [11]. Patients do not always remember what a study is about, and some (including lay people) do not like the idea of being randomised because they think they will not receive the best possible treatment [15, 16].

In order to explore the reasons for taking part, benefits and challenges within the context of the experience of traumatic injury the research question for the study was: what are patients’ experiences of taking part in a trial of standard wound management versus NPWT whilst they are in acute care.

## Methods

This study was embedded in a clinical trial comparing standard wound management with NPWT. Patients were recruited who had an open fracture of the lower limb (where the bone protrudes through the skin). Due to exposure of the wound to air and debris, infection is a risk and early surgical intervention is required to clean the wound and remove damaged tissue. The primary outcome was the Disability Rating Index at 12 months. Consent for the study was taken if the patient had capacity. If it was judged that the patient did not have capacity, either a family or friend acted as personal consultee or a surgeon not directly related to the trial acted as a nominated consultee. After their surgery, when capacity was regained, participants were informed of the study. They were provided with a written information sheet which included a diagram of the NPWT supported by verbal discussion before deciding whether they would like to consent to continue in the study.

The methodology drew on phenomenology using interviews to illicit participants’ lived experience of taking part in the trial [5]. This approach allowed participants to present what it is like to be in the world from their perspective in light of their past history and social context [17]. The aim was to understand what it was like for them to participate in the trial of wound dressings, in the context of an open fracture of the lower limb. To enable participants to express their experience in their own words the interviews were lightly structured around the question: What is it like to take part in the trial? followed up with prompts, such as How did that feel? What did you think?, and tell me a bit more about that.

Under the terms of approval granted by the Local Research Ethics Committee (reference 10/57/20, 6 February 2012), a purposive sample of 20 patients who had consented and one who had declined to take part in UK WOLLF were interviewed from July 2012–July 2013. The participant interviewed who chose not to go into the study had an aversion to undertaking paperwork, due to his experience of school life. On average they were 12 days post first surgical intervention and included a range of ages, gender and experience. All the participants had a severe open fracture of the lower limb and most had skin or muscle grafts. Injuries were sustained at work, at home or from road traffic collisions. The interviews took place within the ward area and were 54 min long on average. The interviews were audio-digitally recorded and transcribed verbatim. Analysis took place drawing together codes derived from meanings inherent in the interviews, drawing them together into categories and themes or ‘structures of experience’ [18]. NVivo 10 a software package was used to help manage the data. Rigour was demonstrated through trustworthiness which included immersion in the data, providing an audit trail with participants’ quotes and reflections in field notes and with peers [19].

## Results

The findings from this study identify the theme of being compromised expressed through the categories of being dependent, being trusting, being grateful and being without experience which reflects the participant’s experience of being asked to be in the UK WOLLF trial [5, 6]. Being compromised in this study was defined as: a way of living that enabled participants to engage in the trial within their current experience of vulnerability as a result of injury. It was expressed through trust in the research as well as the clinical team to do their best and to facilitate their longer-term recovery. They made sense of the treatments within the context of injury and its effect on their life whilst knowing that they lacked understanding and personal experience of the treatments.

### Category: being dependent on others

The participants recognised their vulnerability and were aware of their compromised state at the time of being told about the trial. They noted that they were ‘not quite with it’ (participant 11) and ‘I would not have made much sense’ (participant 8); consequently they were dependent on others to make the decision for them. Those that could remember being told about the trial felt that the intervention was of minimal concern to them as both were used in practice:

I was informed about it on the first day and I can half remember being informed about it because I was on gas and air and morphine, tramadol, I was on all sorts of stuff the first day, but yes I can remember being asked about it. I can remember agreeing to it then but not (being) quite with it. Then they mentioned it afterwards and yes I can’t really see it having that much effect on me. It sounds like they’re both widely used and they both do their job and as long as it does its job I have absolutely no qualms about it. Yes, absolutely no worries about it at all and if it helps discover the best way to sort out open fractures then I’m more than happy to be a guinea pig as it were, that doesn’t bother me. Participant 11

In general, the participants preferred to be involved in the study to the degree in which they were able. Some felt early information helped them to make sense of the study when consent was obtained for continued enrolment in the study at a later stage. The type of wound dressing was considered less important than other therapeutic activity such as being ‘fixed’, but they valued knowing about their treatment decisions. They were aware that often choice is limited and balanced this with the impact on their life:

I would rather that they woke me up and asked me given the choice...as far as I’m concerned there was no invasion of my privacy, it was just to see what dressing worked best it’s neither here nor there as far as I’m concerned. If no-one had ever told me I would have been no worse off, it wouldn’t have affected my life in any way. Participant 15

The low demands on their time and effort made by the study process in relation to activity whilst in hospital and follow-up visits was considered a benefit alongside the ease at which they could withdraw from the study:

It genuinely didn’t bother me about that and I felt free at any point to say no. It was made very clear to me that it was voluntary and I could withdraw at any time so I didn’t have any qualms about it at all…had it been more intrusive or more that they needed to check me every week or something like that, than logistically I wouldn’t have been able to do that. Participant 16

Being dependent on others, due to the severity of their injuries, was accepted as a normal part of care and something they had little control over. The nature of the intervention in this study was acceptable as both were used in routine practice and it was considered a low-risk intervention compared to the invasive surgical interventions that they were receiving. Their views of the study were, therefore, situated within their broader experience of injury. However, they understood that they had limited capacity prior to randomisation but participants generally wished to be involved as much as they were able.

### Category: being trusting

There was an enormous degree of trust invested in the clinical team due to the severity of the nature of the injury, and this was extrapolated to the research team; the research team being integrated into the clinical team looking after the patients’ overall care. Both teams were a source of knowledge and it was assumed that they would know what was best for each individual:

I’m stood here to tell the tale and I’m just glad of that and taking part in any trials or vacuum pumps (NPWT) as such, the decisions were made for me as I wasn’t in a fit state of mind to do that at the time. To be honest, even if I was I would have agreed to it anyway. If they had said to me they had got this and that I would have said to them straight, what do you think? I would have passed the buck back, which was the best one… Participant 3

Trust in the team was based on hope that with the team’s support they had a future. The need to trust and believe in the team overshadowed their feelings about the type of dressing they received:

Bring me out the other side, as long as that happens I’m happy regardless of how they do it. Participant 5

Trust was interlinked with beliefs about the knowledge differential between staff and patients in relation to wound healing. It was felt the clinical and research teams had greater knowledge and, therefore, should be making the decisions. Although those with knowledge of NPWT gained from medical family members would have preferred to have received it as part of their care:

They know what they’re doing and it’s obviously better that they’re doing it and I’m not. Participant 14

The severity of their injury left them feeling emotionally fragile, with strong emotions that they had often never felt before or only when a family member had died. They felt their wounds looked visually horrific and they struggled with pain, lack of mobility and had difficulty imagining how they could live with such injuries [5, 6]. Trust in the team was a way of being hopeful about future recovery. Their own knowledge and skills were limited so they invested their trust in the expertise of the team.

### Category: being grateful

Participants were incredibly grateful for being alive, being saved and felt very lucky. They wished to give something back and being part of a trial was one way of fulfilling this need. Altrusim in the form of the need to help others particularly those of a certain community, such as motorcyclists or the scientific community, were noted:

I thought I felt a bit privileged actually to help in that respect if that’s the case. You need to try and give something back. Yes, I was glad to do it, at the end of the day you have to try and give something back to something that’s been good to you, it’s worth doing isn’t it really and it helps to learn from everyone’s experience. Participant 10

The notion of reciprocity and wanting to give something back is evident in clinical trials [12]. The particpants often had near death experiences and talked extensively of those who had saved them or undertaken extensive surgical interventions to provide them with a chance of walking again [5, 6]. They were aware of the intense activity that have given them a chance of recovery and wanted to support the development of knowledge that might help others. Some also felt a sense of belonging to the biking community and wished to support others who may have a similar accident in the future.

### Category: being without prior experience

It was very clear that despite prior discussion of the treatments participants felt that they had limited understanding of the interventions and would only gain this understanding through experience:

The thing is I have never had this situation before so it’s not like I can say last time I had standard, this time I’ve had vacuum and I found the vacuum (NPWT) is a lot better. I can only tell you what I know and this is all I know. Participant 5

Once experienced there was a strong preference for the NPWT as it was considered to have a multitude of benefits. It was described as clever technology that visibly drew away ‘dirty’ fluids from the body and provided a clean area that protected the wound. It was felt to have an active element leading towards recovery and was psychologically reassuring:

Personally from my viewpoint and psychologically the vac (NPWT) was very good, it made me feel like that was a clean space and nothing was getting in but whether that’s true or not I don’t know, but psychologically looking at it and seeing everything you felt like it was very good. Participant 15

Rather than bandages soaking up all the goo that was coming off to see it being mechanically extracted for me was, as an engineer, more reassuring than just stood there. I wouldn’t like to think that my leg could lay in a puddle of goo when it could be freshly vacuumed, so in a way I found that reassuring. That was good enough for me so there were no issues there. Participant 2

The sense of security that the NPWT provided was within the context of a high level of technological care where another tube could make little difference to participants:

I had a drain from this wound, I had a catheter, I had two cannulas and they were taking blood out of this arm every now and then, I had oxygen, I had an epidural, I had wires coming out of everywhere, it was really strange because I woke up and it was …, well I couldn’t really move but yes so having another tube wouldn’t have really bothered me at all. Participant 11

Concerns were expressed about the pain of dressing removal to stickiness and hairs, sensitive skin, managing the tubes when in bed and when mobilising:

On the flip side, you are very constrained, you are very scared to move, very frightened of interrupting or damaging it, it’s bit like with the neckline anything with tubes and that you don’t want to do too much in case it causes problems. Participant 15

The standard dressing was accepted as just there and as long as it did not cause pain and was cleaned appropriately participants were happy with them:

Yes, it is a worry but I don’t worry about it because I see the nurses do it every other day and as long as I see it being cleaned properly and it is, they’re really thorough and it doesn’t hurt it’s really helped me to think actually it’s alright, it’s just part of my leg at the minute but it will be okay. It’s really good to know. Participant 1

Knowing gained from experience appeared to be important to how participants made sense of the study interventions. As they had no experience of injury or either of the dressings they felt their knowledge of the interventions was limited so taking part in the study was an easy decision. Direct experience of the NPWT led to a preference for this dressing. Experiential knowledge may, therefore, be a useful additional tool for informing trial participants about interventions.

Being compromised meant that participants were dependent on others to act on their behalf until they felt well enough to consent for themselves. In this acute period they invested a high degree of trust in the team, for clinical decisions as well as research, but also in relation to hope for their future recovery. They wished to be involved in trial decisions but were aware that they were compromised by the impact of injury and treatment diminishing their ability to function normally. Wound dressings were considered a minor aspect of their care in relation to the enormity of other therapeutic treatments. Being grateful for their care, altruism based on helping others and the minimal trial requirements were facilitators of trial participation. In making sense of the study they were hampered by their lack of understanding and experience of the two treatments but showed a preference for NPWT once experienced.

## Discussion

The findings identified that patients found that being part of a trial was closely aligned to their broader experience of injury. As the clinical and research teams are integrated, much of the faith in the clinical team was extrapolated to the trial and, from the patients’ perspective, the trial interventions were an integral part of their clinical care. This was expressed though their trust in the team as a whole. Another key finding was their preference for a degree of involvement in the trial despite not necessarily being able to provide informed consent before the interventions took place.

Being compromised by traumatic injury placed participants in a position where their autonomy or freedom to act was constrained by their ability to function and their knowledge about best treatment and care. In recognising their dependency on others, participants placed their trust in the knowledge and skills of the team and their experience so far of being ‘saved’ [5, 6]. Relationships with the clinical team can be crucial with some trial participants taking a ‘leap of faith’ and basing participation on feelings about the team rather than information about the study [9]. The context of emergency care may, therefore, constrain individuals’ ability to voluntarily choose to take part in a study but Gillies and Entwistle [20] suggests the emphasis on rational decision making ignores the social context and relational aspects of people’s lives. They suggest a move towards supporting autonomy whilst taking these aspects into account. In this study participants perceived their degree of choice as limited by the emergency nature of their condition also identified by patients with other conditions [9]. Participants were sustained by trust and hope in the team that they would help them recover. Hope has been identified as important in recovery from trauma [21] and may be a way of mitigating uncertainty regarding further loss of a limb, infection or surgery and their ability to return to normal. Uncertainty is often present where, despite a desired outcome, the way to achieve it is not clear [22]. In major trauma often the route to recovery is not straightforward for participants so placing their hope in staff could provide comfort from the emotional turmoil and uncertainty that injury generates [5]. Supporting individual autonomy to make an informed decision regarding participation in a trial, therefore, needs to be balanced with an understanding of the importance of patients’ trust in the team and hopes for future recovery.

The principle of involving participants in the study, even when they did not have the capacity to consent to the study, was important for some patients. Patients in this trial could be enrolled without their prospective informed consent, through personal or nominated consultees, due to their poor physical and mental state. On consenting to continue in the study some would have preferred to be involved prior to randomisation also noted in other trauma studies [13]. However, overall they did not experience this decision as morally problematic as they felt they needed help from professionals and that wound dressings were minor compared with the importance of being ‘fixed’. Meaningful involvement of patients in health care decisions is often a complex process influenced by many factors [23]. ‘Inclusionary consent’, a term used to identify gaining permission within the limits of participants’ capacity whilst formal agreement is gained from others [24] as used in dementia care may be a helpful way forward. Further consideration of how patients can be involved despite lacking capacity to provide informed consent is recommended.

In making a decision to take part in the trial the participants were constrained by their ability to understand and make sense of the interventions. They were influenced by altruism, the personal benefits of taking part, as identified by others [12, 13], and the uncontroversial nature of the intervention and experience of the team. The degree of knowledge required for participants to understand a study and make sense of the study in light of their experience and current context is difficult to ascertain. Understanding can be affected by factors such as literacy, comprehension and inferences gleaned from staff. A good decision should be more than understanding but include participants experience of what is important to them and the impact on their life [20]. In this trial the decision appeared to be easy to make as the interventions were considered minor in relation to the enormity of other treatments and were both used in everyday practice so the degree of ‘risk’ was minimal. They did not mind being part of an experiment, a ‘guinea pig’, whereas in a study of two types of metalwork there were concerns about the interventions not being tried and tested [13]. On reflection, and from those who had experience of the NPWT, there was a realisation that they could never truly ‘know’ the interventions without having any experience of them, also noted in ankle injury [11]. For some there was a sense of therapeutic misconception; they felt that the surgeon had chosen the best treatment for them, had limited understanding of the interventions, risks and randomisation as found in other trauma studies [13]. In general, they placed their trust in the team that they would not consider them for a study that would harm them. Although sometimes there was surprise they had been put into a study, there was no sense of randomisation as unacceptable as identified in other studies [15, 16]. A strong preference for NPWT once experienced also reflects feelings of trust and a sense of security in the use of technology as noted in critical care [25]. Patients lack experiential knowledge that they feel would help decision making and the boundary between clinical and research interventions is often unclear. It is therefore recommended that clinical and research staff work closely on the presentation of research material whilst being aware of patient perspectives.

Limitations of the study are that the sample was not chosen to be ethnically diverse. Broader understanding of cultural experiences may have identified variation in the trust of the scientific community and the need for a range of language-appropriate media [21]. Due to the impact of injury the interviews were based on recall of experience; different methodological approaches might be able to capture thoughts, feeling and interactions at the time of being informed about the study and how perspectives change over time. In order to consider the transferability of study findings to other areas a clear account of the sample, methods and use of participant quotes to support the findings are presented. However, we do not know if wound dressings would be a minor element of care where the impact of injury is less severe or the dressings were not already used in current practice.

## Conclusion

Within the theme of being compromised, participants in this study made sense of trials within a context of vulnerability. They reported their feelings of dependency on others, their lack of knowledge and their need to trust and invest hope in the team for their future recovery. However, they expressed the desire to be involved in the research decision-making, even if they could not provide formal consent prospectively. This study suggests that researchers involved in trials of emergency interventions should: (1) be aware that trust and hope in the team are an important part of patient experience of recovery from injury but a balance is required between supporting their hope and the requirement for informed decision making, (2) involve the potential participant in the trial to the limit of their capacity, even if formal consent is not possible and (3) work closely with the clinical team when presenting trial information and be aware that patients will not necessarily separate clinical interventions from research interventions.

## Abbreviations

ISRCTN: International Standard Randomised Controlled Trials Number
NPWT: Negative Pressure Wound Therapy
NVivo 10: is a software package that helps in the management of qualitative data

## References

[1] Costa ML, Tutton E, Achten J, Grant R, Slowther AM (2017). Informed consent in the context of research involving acute injuries and emergencies. Bone Joint J.

[2] Lawton J, Hallowell N, Snowdon C, Norman JE, Carruthers K, Denison FC (2017). Written versus verbal consent: a qualitative study of stakeholder views of consent procedures used at the time of recruitment into a peripartum trial conducted in an emergency setting. BMC Med Ethics..

[3] Staniszewska S, Boardman F, Gunn L, Roberts J, Clay D, Seers K, Brett J, Avital L, Bullock I, O’Flynn N (2014). The Warwick patient experiences framework: patient-based evidence in clinical guidelines. Int J Qual Health Care..

[4] CG138 NCG (2012). Patient Experience in Adult NHS Services: Improving the Experience of Care for People Using Adult NHS Services.

[5] Costa ML, Achten J, Bruce J, Davis S, Hennings S, Willett K, Petrou S, Jeffery S, Griffin D, Parker B, Masters J, Lamb SE, Tutton E, Parsons N. UK wound management of open lower limb fractures (UK WOLLF) a randomised controlled trial and health economic evaluation of standard wound management versus negative pressure wound therapy in the treatment of adults with an open fracture of the lower limb. Health Technol Assess. 2018. in press. 10.3310/hta22730 PMC6322061 30573002

[6] Tutton E, Achten J, Lamb SE, Willett K, Costa M, on behalf of the UK WOLLF Research Collaborators (2018). A qualitative study of the experience of an open fracture of the lower limb in acute care. Bone Joint J..

[7] Costa ML, Achten J, Bruce J, Tutton E, Petrou S, Lamb SE, Parsons NR, UK WOLLF Collaboration. Effect of negative pressure wound therapy vs standard wound management on 12-month disability among adults with severe open fracture of the lower limb: the WOLLF randomised clinical trial. JAMA. 2018;319:2280–88. 10.1001/jama.2018.6452 PMC6583504 29896626

[8] Canvin K, Jacoby A (2006). Duty, desire or indifference? A qualitative study of patient decisions about recruitment to an epilepsy treatment trial. Trials.

[9] McCann SK, Campbell MK, Entwistle VA (2010). Reasons for participating in randomised controlled trials: conditional altruism and considerations for self. Trials..

[10] Harrop E, Noble S, Edwards M, Sivell S, Moore B, Nelson A, Group FTM (2016). I didn’t really understand it, I just thought it’d help’: exploring the motivations, understandings and experiences of patients with advanced lung cancer participating in a non-placebo clinical IMP trial. Trials.

[11] Keene DJ, Mistry D, Nam J, Tutton E, Handley R, Morgan L, Roberts E, Gray B, Briggs A, Lall R (2016). The ankle injury management (AIM) trial: a pragmatic, multicentre, equivalence randomised controlled trial and economic evaluation comparing close contact casting with open surgical reduction and internal fixation in the treatment of unstable ankle fractures in patients aged over 60 years. Health Technol Assess.

[12] Locock L, Smith L (2011). Personal benefit, or benefiting others? Deciding whether to take part in clinical trials. Clin Trials.

[13] Huxley C, Achten J, Costa ML, Griffiths F, Griffin XL (2016). A process evaluation of the WHiTE two trial comparing total hip arthroplasty with and without dual mobility component in the treatment of displaced intracapsular fractures of the proximal femur: can a trial investigating total hip arthroplasty for hip fracture be delivered in the NHS?. Bone Joint Res..

[14] Lidz CW, Appelbaum PS, Grisso T, Renaud M (2004). Therapeutic misconception and the appreciation of risks in clinical trials. Soc Sci Med..

[15] Featherstone K, Donovan JL (2002). ‘Why don’t they just tell me straight, why allocate it?’ The struggle to make sense of participating in a randomised controlled trial. Soc Sci Med..

[16] Robinson EJ, Kerr CE, Stevens AJ, Lilford RJ, Braunholtz DA, Edwards SJ, Beck SR, Rowley MG (2005). Lay public’s understanding of equipoise and randomisation in randomised controlled trials. Health Technol Assess..

[17] Heidegger M (1962). Being and time.

[18] Van Manen M (1990). Researching lived experience, human science for an action sensitive pedagogy.

[19] Lincoln YS, Guba E (1985). Naturalistic inquiry.

[20] Gillies K, Entwistle VA (2012). Supporting positive experiences and sustained participation in clinical trials: looking beyond information provision. J Med Ethics.

[21] Tutton E, Seers K, Langstaff D (2012). Hope in orthopaedic trauma: a qualitative study. Int J Nurs Stud..

[22] Morse JM, Penrod J (1999). Linking concepts of enduring, uncertainty, suffering, and hope. Image J Nurs Sch.

[23] Smith SK, Dixon A, Trevena L, Nutbeam D, McCaffery KJ (2009). Exploring patient involvement in healthcare decision making across different education and functional health literacy groups. Soc Sci Med..

[24] Dewing J (2008). Process consent and research with older persons living with dementia. Res Ethics Rev..

[25] Stayt LC, Seers K, Tutton E (2015). Patients’ experiences of technology and care in adult intensive care. J Adv Nurs.

